# Necrotizing Fasciitis Versus Pyoderma Gangrenosum: Securing the Correct Diagnosis! A Case Report and Literature Review

**Published:** 2011-05-13

**Authors:** Kamal Bisarya, Silvan Azzopardi, George Lye, Peter James Drew

**Affiliations:** Welsh Centre for Burns and Plastic Surgery, Morriston, Swansea

## Abstract

**Objective:** To highlight the key differences in history, examination, and management of pyoderma gangrenosum and necrotizing fasciitis and to outline the importance of distinguishing these 2 conditions. **Method:** We present a case report of a gentleman with a background of ulcerative colitis having a 1-week history of an erythematous wound and localized abscess to the right leg that failed to respond to antibiotic treatment and later on to surgical debridement of a presumed necrotizing fasciitis. Following referral to our plastic surgery unit, a diagnosis of pyoderma gangrenosum was made and this was confirmed following a response to steroid therapy within 48 hours. A literature review of pyoderma gangrenosum cases misdiagnosed for necrotizing fasciitis was carried out to compare and contrast pitfalls in misdiagnosing these 2 conditions. **Results:** Literature review of 10 cases confirmed the association of pyoderma gangrenosum with inflammatory bowel disease, hematological disease, and surgical trauma. The presence of necrotic tissue in a pyoderma gangrenosum lesion can be a diagnostic pitfall; although blood and tissue culture investigations are usually negative in pyoderma gangrenosum, this may not always be the case. Inflammatory markers can be significantly high in pyoderma gangrenosum and pyrexia is not a feature limited to necrotizing fasciitis. **Conclusions:** Inappropriate surgical debridement of pyoderma gangrenosum can cause rapid extension of the lesion by enhancing the posttraumatic response and lead to potential reconstructive challenges with psychological repercussions. On the contrary, treating necrotizing fasciitis with immunosuppressive therapy may worsen the condition. The importance of understanding the pathogenesis, clinical features, and management of both conditions cannot be overemphasized.

Pyoderma gangrenosum (PG) is a dermatological condition that can be easily mistaken for infective conditions including necrotizing fasciitis (NF). We would like to use our case report as an example of initial wrong diagnosis and inappropriate management of PG followed by a literature review of PG cases misdiagnosed for NF. The key differences in history, examination, and management of these 2 conditions will be highlighted

## METHODS

We located all articles reported in the English language where PG was mistaken for NF by conducting a literature search on PubMed using the following key words: necrotizing fasciitis, pyoderma gangrenosum, misdiagnosis, and case reports. Nine cases were identified and analyzed to synthesize our data, which were summarized in table format to highlight the chronology of clinical progression in each case.

## CASE REPORT

**Figure 1:**
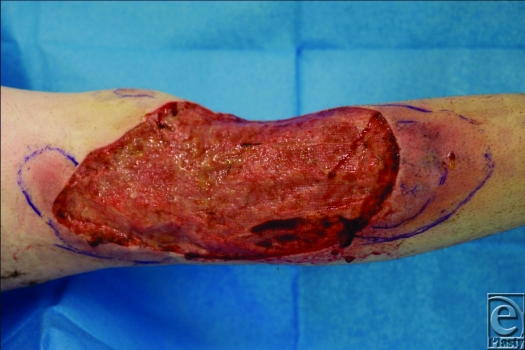
Lower limb highlighting worsening of *Pyoderma gangrenosum* postdebridement.

J.W. was a 33-year-old male IT analyst whose medical background included ulcerative colitis for which he was on pentase and predfoam enema. He was referred by the general practitioner (family physician to the emergency department with a 1-week history of an erythematous wound to his right lateral leg with no improvement on oral flucloxacillin. Hospital review revealed development of a “localized abscess,” and associated inflammatory markers included a White cell count (WCC) 13.4 and Creactive protein (CRP) 179. Subsequently the patient was admitted by the orthopedic team and treated with intravenous antibiotics (benzylpenicillin, flucloxacillin, and metronidazole). Four days later he underwent incision and drainage where the operative findings confirmed “blood stained pus.” Two days later, an increase in the WCC to 20.3 and CRP to 283 was observed and a further debridement of a 25 × 15-cm area was undertaken where “a bubbly appearance of the margins, extensive erythema and necrosis of the skin distal to the wound, discharge and fat necrosis” were noted. Histology confirmed inflammation with necrosis of the tissues, but Gram stain showed no specific organism and wound swabs only grew coagulative negative *Staphylococcus*. The case was discussed with the microbiologists who recommended treating the patient with a provisional diagnosis of NF. However, as there was no clinical improvement at this stage, he was referred to our burns and plastic surgery department. An assessment in theatre found “purple borders around the wound, and subcutaneous pockets of pus which discharged on palpation.” Histology confirmed “an ulcer and abscess with acute inflammation and fibrosis and granulation tissue which extends to the margins with no vasculitis or neoplasia.” Microbiology failed to show any growth. Based on these clinical findings, histology, and microbiology, a diagnosis of PG was made and the patient was commenced on prednislone 40 mg. Subsequent review revealed improvement within 48 hours with no extension of the wound, and the patient went on to have a split-skin graft to cover the previous defect with subsequent full recovery (Fig 1).

## RESULTS/ANALYSIS OF THE LITERATURE

Table 1 summarizes the English literature review of cases where PG has been misdiagnosed for NF. The association of PG with a medical or family history of inflammatory bowel disease (case Nos. 1, 2, 3, 6, 7, 10), surgical trauma and/or worsening after surgery or even intravenous cannulation (case Nos. 1 2, 3, 5, 6, 7, 8), and an association with hematological disease (case No. 9) can all be demonstrated. All cases of PG were initially misdiagnosed as being infective conditions ranging from abscess to cellulitis or NF and were accordingly treated with antibiotics, surgical debridement including multiple debridements (case Nos. 6,7), repeated excision for histology sampling (case Nos. 9), and even digit or partial toe amputations (case Nos. 1, 3).

The presence of necrotic tissue can be a diagnostic pitfall, this is seen, for example, in case No. 9, where the patient at one point in his clinical course had intense pain, pyrexia, inflamed skin, and necrotic tissue favoring NF as a diagnosis but preoperatively superficial and muscular fascia appeared healthy. Necrotic tissue was also seen in case Nos. 3, 5, and 7. The presentation of case No. 4 also is misleading, where a 58-year-old woman presents with extreme right breast pain, swollen red skin that rapidly progresses to a black discoloration within a few hours and eventually does require excision of necrotic areas and healing by secondary intention. In any case the presence of necrotic tissue makes the diagnosis of PG more obscure.

In the cases listed in Table 1, while blood culture and/or tissue culture investigations including wound swabs yielded no positive microbiology in several instances—thus excluding an infective etiology—this was not always the case. Case No. 6 grew cultures that isolated *Staphylococcus aureus* with mixed growth from an ankle wound that initially presented with ulcers, erythema, and abscesses but subsequently responded to surgical debridement only to recur again and be misdiagnosed as NF. This same patient developed violaceous ulcers in an SSG donor site that grew mixed growth of skin commensals; in both instances the positive cultures were more likely to be colonizers as this was a case of PG not NF. In case No. 3, *Staphylococcus aureus* and *Enterobacter cloacae* were grown from a postoperative wound, which had pustules and areas of necrotic tissue but subsequently all cultures were negative while pustular and vesicular lesions were noted. Case No. 7 also presents a similar diagnostic dilemma between NF or colonized or infected PG with necrotic tissue.

The biochemical markers in the PG cases reported earlier do not always follow a consistent pattern, CRP ranges from 14.5 (case No. 6) to 269 (case No. 9) were noted in active stages of the disease and similarly white blood cell count has been reported as normal (case No. 6) or raised (case No. 9). Pyrexia is not a sign restricted to NF and this has been noted in case Nos. 5, 8, 9, and 3 the latter as high as 39.8°C.

The time scale between initial presentation to making a definite diagnosis of PG and initiating appropriate treatment ranged from 2 days to 4 weeks, although case No. 3 had an approximate 14-week delay in having a correct diagnosis, this was established on a graft donor site that developed pustular lesions and not on the initial operative site, which by then had healed. In these cases, the initial response to treatment of PG was noted as early as a few hours up to within 3 days of treatment. The delay in clinching a proper diagnosis shows the extent of the diagnostic dilemma at times.

## DISCUSSION

### Pyoderma gangrenosum versus necrotizing fasciitis

In 1930, Brunsting et al^1^ reported clinical and experimental observations in 5 cases of pyoderma (ecthyma) gangrenosum in adults, the condition erroneously being thought to be a streptococcal or staphylococcal infection.^1^ A number of cases of PG have been reported in the literature (Table 2) to be misdiagnosed for NF, despite the fact that the pathophysiology and management of both conditions are totally different.

Pyoderma gangrenosum is a rare, primarily sterile inflammatory neutrophilic dermatosis^2^ that may present in ulcerative, pustular, bullous, and vegetative forms.^3^ This condition has an incidence of 3 to 10 per million per year^4^ and it can affect all age groups with the youngest documented patient being a 3-week-old newborn.^5^ Women are more often affected than men and the peak incidence for adult females is in the third and fourth decades and for males it is in the fifth decade.^6^ It is found in up to 5% of patients with ulcerative colitis and in up to 1.5% of patients with Crohn disease.^7^,^8^ It is known to be associated with systemic disease such as rheumatoid arthritis, ankylosing spondylitis, leukemia, myelodysplasia, lymphoma, and other miscellaneous disorders such as sarcoidosis, human immunodeficiency virus infection, and systemic lupus erythematosus.^7^,^9^ Up to 40% of patients may have PG lesions in the absence of associated disease^4^ and history of pathergy whereby lesions are precipitated by trauma is reported in 25% of patients.^4^

One of the challenges in making a correct diagnosis of PG lies in the fact that it relies heavily on clinical signs as there are no immunofluorescent markers, and in addition, histopathology is nonspecific and changes with progression of the lesion.^2^,^7^ The morphological evolution of PG lesions encompasses perivascular lymphocytic infiltration associated with endothelial swelling, while ulceration, areas of necrosis, infarction, and abscess formation may be found in the later stages.^10^ Pyoderma gangrenosum lesions have a predilection for the pretibial areas, although they can affect any other body site including oral, genital mucosa, and viscera such as lung or spleen.^2^ A typical lesion would be a follicular pustule that ulcerates and is surrounded by erythematous, edematous skin. The ragged, undermined, and violaceous or bluish ulcer border are distinctive of this condition and key in making the diagnosis.^2^,^6^ The 6 disease categories that are included in differential diagnosis of PG are infective such as NF, vascular occlusive or venous disease, vasculitic processes such as Wegener's, malignancy such as lymphoma or leukemia, pustular drug reactions, and exogenous tissue injury such as insect bites.^2^,^11^

Necrotizing fasciitis is a relatively uncommon, potentially fatal, poly- or monomicrobial infection predominately involving subcutaneous fat, superficial fascia, and deep fascia. This condition not only is limited to susceptible patients such as the malnourished, obese, immunocompromised, diabetic patients or those with malignant disease (type1 NF) but may affect previously healthy individuals who report a history of only minor trauma, such as after penetrating insect bites (type 2 NF).^12^,^13^ Necrotizing fasciitis has been documented to occur in virtually all anatomical regions of the body but has a predilection for the extremities, the perineum following an ischiorectal or pilonidal abscess and the abdominal wall such as after abdominal surgery.^12^ In a study of 83 patients with NF treated over a 17-year period,^14^ the commonest pathogenic aerobes were *Staphylococcus aureus* followed by *Escherichia coli* and then group A streptococci. The predominant anaerobes were *Peptostreptococcus* spp, *Prevotella*, *Porphyromonas*, *Bacteroides fragilis* group, and *Clostridium* spp. Anaerobes outnumbered aerobes and certain microorganisms were associated with specific conditions such as *Pseudomonas* with immunosuppression and malignancy, *Bacteroides* spp with diabetes, and clostridium with trauma.^14^ Necrotizing fasciitis may present with a patchy discoloration of the skin and ill-defined erythema that can rapidly be followed by a tense oedema while vesicles and bullae may appear together with necrosis. Pain is generally out of proportion to the physical findings while the patient can rapidly become septic and systemically unwell.^12^,^15^

Diagnosis of NF is guided by clinical features, blood cultures, and Gram stain to identify causative organisms,^16^ together with scoring tools such as the Laboratory Risk Indicator for Necrotizing fasciitis, which takes into account CRP, serum Na^+^, Cr, glucose, WCC, and hemoglobin to calculate a low, intermediate, or high risk of NF.^17^ Magnetic resonance imaging excludes NF if deep fascial involvement is not demonstrated; however, this modality of investigation has its limitations as primarily sensitivity is greater than specificity and there are time constraints within which to secure the diagnosis.^18^ The gold standard for diagnosis of NF is direct inspection of fascia at surgery^16^ and histology that will reveal minimal change in epidermis, a lymphohistiocytic infiltrate in dermis, suppuration, with necrosis of superficial fascia and blood vessel thrombosis and oedema in the fascial planes.^12^

Clearly these 2 conditions have different pathophysiology and clinical features; Table 1 compares and contrasts PG with NF to highlight salient points that may prevent misdiagnosis of either condition.

## CONCLUSION

Although several of the clinical features outlined earlier can be identified in the literature review cases, the clinical presentation of either condition may be atypical and this, coupled with confounding laboratory or histological results, creates diagnostic pitfalls.

The importance of understanding the pathogenesis, clinical features, and management of both conditions cannot be overemphasized. Aggressive management of PG with antibiotics and extensive surgical debridement not only will lead to large tissue defects with potential reconstructive challenges^7^ but can cause rapid extension of the lesion by enhancing the posttraumatic response.^19^ On the contrary, treating NF with immunosuppressive therapy may worsen the condition and some authors recommend corticosteroid therapy in association with antibiotics in dubious cases.^19^ The psychological repercussions of misdiagnosis and incorrect management especially that leading to tissue loss are not to be underestimated.^20^ This article highlights how important it is to apply the basic principles of surgery into clinical practice and formulate a diagnosis on the basis of proper history, clinical examination, and investigations; it is crucial to reconsider the preliminary working diagnosis if an expected clinical response is not observed.

## Figures and Tables

**Table 1:** Literature review of cases of pyoderma gangrenosum misdiagnosed for necrotizing fasciitis

N^0^	Author	Age, y; sex	Affected area	PMH	Initial diagnosis	BC TC	Histology	1° Mx	2° Diagnosis /2° Mx of PG	Response time to Rx
1	Tay YK^7^ 1998	38; F	Erythematous painful plaques left palm/middle finger. (similar history 2 y before 2° to blunt trauma) Sites of intravenous (IV) cannula (later)	Crohn disease diagnosis of 3 y prednislone discontinued	1. Cellulitis	-ve	Left-finger ulcer Nonspecific neutrophilic dermal infiltrate	1. Anitbiotics	≥ 1 wk after day 1 MPSS for 3/7 prednislone sulfasalazine	12 h
					2. Necrotizing fasciitis			2. Debridement		
								3. Amputation of digits		
2	Bennett^21^ 1999	25; F	Febrile with cellulitis to skin of MCP joints postrevision arthroplasty and removal of silicone implants bullae; ulcers with purple hue 2/52 later	Juvenile RA Crohn disease	1. Cellulitis	-ve	Not reported	1. IV antibiotics	2/52 after day 1 MPSS for 3/7 Prednislone Po	24 h
					2. Abscess			2. Drainage		
								3. Debridement		
3	Bennett^21^ 1999	32; F	Infected tarsal tunnel release wound right foot Cellulitis Pustules/necrotic tissue (later) SSG donor site – pustular lesions (14/52 later)	Nil	1. Abscess	+ve -ve*	Not reported	1. Antibiotics	14/52 later Prednislone PO	Gradual over 3/12
					2. Cellulitis			2. Debridement x8		
								3. BKA proposed		
								4. HB oxygen		
								5. SSG with 90% take		
								6. Partial toe amputation		
4	Waterworth^22^ 2004	58; F	RT breast, extreme pain, swollen, red skin that discolored black in a few hours. Rapid progress	Ulcerative colitis (years) On Prednislone DVT (1/52 ago)	Abscess hematoma **2**° to warfarin	-ve	Carried out but not reported	Surgical exploration Later excision of necrotic skin and healing by 2° intention	2/7 after day 1 Cyclosporin A ↑ Prednislone dose	Time scale not Specified
			No history of trauma or surgery	Varicose V On warfarin	Nec Fasciitis					
5	Mahajan^23^ 2005	42; F	Inflammation skin necrosis post lap chole.	Nil No disease associated with PG	Nec Fasciitis	-ve	Neutrophilic dermatitis	1. Anitbiotics	4/52 later MPSS for 3/7 Oral Steroids Mycophenolate	Few hours
			IV cannula sites blistered with abscess				No fascial necrosis	2. Debridement		
			Skin graft edge necrosis/ulceration (4/52 from day 1)					3. Vac dressing		
								4. Skin Grafting		
6	Chia^9^ 2008	44; M	Ankle ulcers, erythema/abscess that heal Readmission with necrotizing fasciitis/pyrexia/unwell Crusting, pustules, violet erythema donor site of SSG area; left thigh 3-4/52 post-SSG	Ulcerative colitis diagnosis 8 y Not on Rx in remission	1. Abscess Ankle	†**+ve**	From SSG donor site Neutrophilic dermatitis Perivascular plasma white blood cell infiltrate	1. Abscess excised Response to antibiotics	Prednislone	72 h
					2. Graft failure and prompt dermatology review			2. Necrotizing fasciitis ankle and more antibiotic/debridement		
								3. SS G to ankle		
7	Barr^24^ 2008	70; F	Erythema pus neck 2/52 after BCC excision	History of GI Sx Niece Crohn disease	1. Cellulitis	-ve **+ve**	Ulcers necrosis‡ inflammation consistent with necrotizing fasciitis	1. Antibiotics	≤ 1 wk after day 1 IV steroids	7 d
			Dusky necrosis at CVP line/chest drain site		2. Necrotizing fasciitis			2. Debridement multiple		
					3. Septic Shock			3. ITU administration with shock		
8	Jaivsminsayna^25^ 2009	56; M	Postoperative appendicectomy wound with pain, discharge, and ulceration	-ve Autoimmune markers	Necrotizing fasciitis with 2° sepsis	-ve	Histology consistent with necrotizing fasciitis	1. Anitbiotics	IV steroids	Instant
			Later—nodules/ulceration at cannula site					2. debridement		
								3. ITU care		
9	Ayestaray^19^ 2010	64; M	Cellulitis right arm initially but developing central necrosis within first week suggesting necrotizing fasciitis	Vaquez disease	Cellulitis necrotizing fasciitis	-ve	Reported to favor PG	1. IV antibiotics	Time scale not specified IV steroids	48 h
								2. Repeat Excision (Inflammation worse post surgery)		
								3. Thin SSG with mesh		
10	Our case 2010	33; M	1-wk history of erythematous wound and localized abscess to right lateral aspect leg	Ulcerative colitis	1. Abscess	-ve	Ulcer, abscess, and inflammation with no vasculitis nor neoplasia	1. Antibiotics PO/IV	˜ 2/52 after day 1 Prednislone PO	48 h
					2. Necrotizing fasciitis			2. Debridement		

Mx indicates managment; PMH, past madical history; MPSS, methylprednisilone (sodium succinate); BKA, below knee amputation; BC, blood culture; TC tissue culture; ITU, intensive therapy unit; RT, right; CVP, central venous pressure.

* Positive from wound 9 days postoperatively: *Staphylococcus aureus* and *Enterobacter cloacae*. Diagnosis of infection but pustules and areas of necrotic tissue noted. Subsequently all cultures were negative; pustular and vesicular lesion present.

†Wound culture from ankle the initially effected site isolated *Staphylococcus aureus* with mixed growth but more likely to be colonizers. Inflammatory pustules in SSG donor site confirm neutrophilic pus; mixed growth of skin commensals in wound culture but no evidence of bacterial infection—diagnosis of pustular PG.

‡Although first histology report confirmed ulcers, inflammation, and necrosis consistent with necrotizing fasciitis, the second histology sample taken after neck debridement was reported as ulcerated skin with exuberant dermal and subcutaneous inflammation and necrosis with bacteria in necrotic areas. This could still be necrotizing fasciitis but could also be colonized or infected PG

**Table 2:** Comparison of pathology and clinical features of pyoderma gangrenosum and necrotizing fasciitis

Pyoderma gangrenosum	Necrotizing fasciitis
Pathology	
Noninfectious neutrophilic dermatosis	Necrotic soft tissue infection
Dermis involved	Fascia and subcutaneous fat involved
May have necrotic areas	Will have necrosis
Clinical	
Strong link with inflammatory bowel disease	No association with inflammatory bowel disease
Slower progression within days	Rapid progression
Does not resemble cellulitis	Can resemble cellulitis in early stage
Violaceous ulcer edge is typical	Violaceous ulcer edge not a typical feature
Unlikely to develop sepsis	Septic picture can develop
Unlikely to require ITU care	Likely to require ITU care
Worsens with surgery	Responds to surgery
Fascial planes have normal resistance to dissection	Lack of resistance to blunt dissection of fascial planes
Responds to immunosuppressive therapy	Worsens with immunosuppressive therapy
No response to antibiotics and worsens with surgery	Should respond to antibiotics and surgery
Usually negative blood and tissue cultures	Usually positive blood and tissue cultures

ITU indicates, Intensive Therapy Unit.
